# Finding the Optimal D/H Ratio for an Enclosed Urban Square: Testing an Urban Design Principle Using Immersive Virtual Reality Simulation Techniques

**DOI:** 10.3390/ijerph16050865

**Published:** 2019-03-09

**Authors:** Jaecheol Kim, Seungnam Kim

**Affiliations:** 1 Department of Urban Planning, Gachon University, Seongnam 13120, Korea; jaecheol@gmail.com; 2 Department of Urban Design and Studies, Chung-Ang University, Seoul 06974, Korea

**Keywords:** urban public squares, enclosure, D/H ratio, virtual reality, visual assessment survey

## Abstract

Using immersive virtual reality (VR), this study examined the D/H ratio principle (where “D” means the depth or width of a public space and “H” means the height of its surrounding buildings.) that urban design theorists have suggested as an important design principle for public spaces. The authors built VR models of eight enclosed urban public squares with different D/H ratios ranging from 1/3 to 6/1. They then carried out an experiment in the form of a visual assessment survey using the VR models with 69 university students majoring in urban planning and compared the optimal D/H ratios from the experimental results with those proposed by urban design theorists. The statistical analysis of the experimental results revealed that the optimal D/H ratios for most qualities of public squares are larger than the optimal ratios claimed by theorists.

## 1. Introduction

As shown in Dempsey et al.’s claim that an attractive public realm is one of the important physical factors influencing urban social sustainability ([1], p. 3), the design of urban public spaces influences people’s social lives [2] and, consequently, the social sustainability of a city. However, regardless of the importance of urban public spaces, many design principles and theories for urban spaces have been based on the intuition or personal experiences of urban theorists rather than on objective evidence (p. 3 [3]). For example, Sitte’s famous works suggesting various principles to design good urban spaces are mainly based on his personal observations and interpretations, although they are insightful [4]. Of course, the experience and insight of urban theorists are valuable resources for the field of urban design, but their theories would be more persuasive if they were supported by objective evidence explaining the causal relationship between the theories and their effects.

As an effective research method to obtain objective evidence for such causal relationships, Yin suggests case studies and controlled experiments ([5], p. 9). Among the two methods, case studies have been widely used in the urban design field, but controlled experiments have not, because it is difficult to carry out controlled experiments in urban settings as people continue to live in the studied spaces and experimentation in such spaces is costly and time-consuming [6]. Case studies are a powerful research tool to analyze the complex urban environment. However, if controlled experiments could be conducted properly, they would be an important complementary resource that could make urban design theory and practice more robust.

Some researchers who felt that there was a necessity for an experimental approach to the urban design field have conducted “pseudo-experiments” or “quasi-experiments” in real urban spaces as a substitute for controlled experiments [5,6], in which they compare several places with similar conditions [2] or compare an urban environment before and after significant changes [7]. As a similar and recent approach, “urban prototyping,” reflecting both tactical urbanism and digital technology, is emerging as a useful method for the rapid testing of new ideas in real urban spaces [8]. However, these efforts in real urban settings have a pitfall, as it is almost impossible to exclude influences by external factors. Other researchers have used 2D visualization methods of urban spaces (in this paper, 2D visualization methods include the perspective projection of the three-dimensional environment onto two-dimensional papers or screens), such as sketches, photographs, videotaping, or computer simulations, to analyze user perceptions of and responses (preferences) to urban spaces [9,10,11,12,13]. Visual assessment methods using 2D images as a substitute for a real urban space have been used as an important tool both for research and practice, but they also have limitations related to the visual distortion that occurs when converting 3D spaces to 2D screens or paper [10,14]. In contrast, virtual reality (VR) technology, particularly immersive VR, is expected to contribute to overcoming such limitations of previous studies by allowing users to experience spaces similar to reality and by enabling researchers to design virtual environments and perform controlled experiments as intended [15,16]. VR technology has already been explored actively and widely in the field of urban planning and urban design for practical and academic purposes.

One of the most promising uses for VR is as an effective communication tool for collaborative design development [17] and public involvement [14,18,19]. A recent project by the Virtual Reality Design Lab of the University of Minnesota is a good example; this is a project to develop a social VR system for architectural design that allows multiple people to view and discuss the same virtual space at the same time [20]. Another promising use of VR in urban design is its use as a research tool for controlled experiments as the authors have suggested above. Many researchers have already used VR as a tool for experiments [16,21,22,23], and some have conducted experiments using VR to test urban design theories or principles [23,24]. However, many urban design theories have not yet been fully tested, and experimental verification using VR is still necessary for such theories.

This study aims to demonstrate how VR could be utilized as a research tool to test urban design theories and principles. In particular, the authors conducted an experiment to test the D/H ratio principle, which is a well-known urban design principle that many urban design theorists have proposed for public spaces, including streets and urban squares [4,6,13,25,26]. The study contains a literature review on related issues and an explanation of the experiment conducted. In the literature review section, the authors review existing literature on the pros and cons of 2D visualization in the field of urban design, the uses of VR in the field, and the D/H principle. In the experiment section, the authors explain the research design of the experiment (a visual assessment survey with VR) and analyze the results.

## 2. Literature Review

### 2.1. Use of 2D Visualization Methods in Urban Design

Traditional 2D visualization tools widely used in the urban design field include pen-paper sketching and photographs (or video clips) ([14], p. 190). Among these, pen-paper sketching, the most traditional, has its own pros and cons. The pros of pen-paper sketching include rapid visualization of both existing and imagined urban environments and three-dimensional representation using the perspective representation method. However, its constraints include inaccuracy in expression (p. 191 [14]), limited viewing angles, and perspective projection distortion.

As a research tool for urban design, photographs have advantages such as increasing the effectiveness of the interaction with an audience including non-experts (it might be more effective than pen-paper sketching) and a high degree of realism ([14], p. 195). Hence, photographs or video records of real urban environments have been used in numerous urban studies [11,12,13]. For example, Nasar compared the visual preferences of students in Japan and in the United States using videotapes and photo slides of urban street scenes in both countries [11]. Ewing and Clemente used video clips of real urban spaces in their visual assessment survey to measure urban design qualities [13]. However, photographs have the same disadvantages as pen-paper sketching because of the limited viewing angles and visual distortions of projecting 3D spaces onto 2D surfaces. They have additional constraints because the real world, which is represented in photographs or video records, has not been created for a specific experiment. Subsequently, it is difficult to control the influence of other factors that are included in the photographs but are not of interest to the researchers (p. 65 [27]) To overcome this problem, photo montage techniques that synthesize the desired images are widely used, but their application is limited ([22], p. 60).

Some of the problems of using sketches and photographs can be addressed by utilizing computer graphic simulations. Computer graphic simulations can improve the accuracy problem of sketching, and they can overcome the difficulties in controlling the influence of the unnecessary factors of photographs. For example, Im and Kim used computer graphic simulations to investigate the effectiveness of design elements for the urban design qualities of urban public squares [9,10]. However, the problems of limited viewing angles and visual distortions remain, and VR technology can contribute to solving these problems.

### 2.2. Use of VR in Urban Design Studies

VR, in the broad sense, includes not only immersive VR but also non-immersive VR that projects 3D simulation models onto a 2D plane [28,29]. In addition, immersive VR includes VR generated with computer graphics or recorded with 360° live cameras [30]. Among its various concepts, in this study, VR is understood as being immersive and computer-generated. Al-Kodmany defined this type of VR as a “3-D, computer-generated synthetic environment or a structure that provides the user with a sense of being immersed in a real world” ([14], p. 197). This definition expresses the two major advantages of immersive and computer-generated VR as a research tool for urban design. First, it provides users with accurate and realistic experiences of spaces. Second, it enables researchers to create any environment to conduct controlled experiments. Because of such advantages, numerous studies on the diverse topics related to VR have been conducted in the area of architecture and urban design.

Many architectural and urban design studies using VR have dealt with the following two research issues. The first group of studies examined the validity and effectiveness of VR as a visualization and communication tool when compared with other tools [14,15,31]. For example, Usman et al. compared three different visual modes (blueprint, first-person view, and VR) in a study on users’ spatial perception [15]. The second group of studies used VR as an experimental method to address practical questions or to test existing urban design theories. For example, to address certain practical questions, Sanchez conducted an experiment using VR to examine the influence of noise on users’ assessment of an urban public space design [16]. Maheshwari et al. used VR in an experiment to examine the influence of street design on people’s choice to use bicycles in Singapore [22]. For recent examples of testing existing urban design theories, the Fisher-Gewirtzman study tested the relationship between visibility and perceived density in the VR environment [21]. Iachini et al. determined reachability and comfort distances between people using an experiment with an immersive VR environment [32]. Furthermore, Tabrizian et al. examined the relationship between the visual permeability of urban green space enclosed by trees and the sense of restoration that people feel [24], and Shushan et al. analyzed the influence of different urban forms on urban imageability [23].

The second group of studies, particularly those that tested unverified urban design theories, is important, as those studies can strengthen the theoretical foundations of urban design and planning fields by providing observable evidence; however, more work needs to be done in this research area. In the next section, the authors introduce the D/H ratio principle, the urban design principle selected for this study.

### 2.3. The D/H Ratio Principle

The authors chose the D/H ratio principle as the urban design principle and tested it using a VR experiment for the reasons discussed below. D/H ratio is the ratio of the width (or distance) of an open space (D), such as public squares and streets, and the height of the surrounding buildings (H). Many urban design theorists have argued that there are proper D/H ratios that can create a “sense of enclosure” for an open space [4,6,25,26,33,34]. For example, Sitte argued that the D/H ratios (in the case of the D/H ratios that Sitte suggested, “H” refers to the height of the principal building of the square) for a square should be between 1/1 and 2/1 ([4], p. 182), Alexander et al. claimed that the D/H ratios for streets should be less than 1/1 ([33], p. 490), Jacobs suggested that D/H ratios should be less than 2/1 [34], and Lynch claimed that the D/H ratios between 2/1 and 3/1 are appropriate, particularly for enclosed open spaces ([6], p. 158). Yoshinobu argued that an adequate balance between height and space exists when the D/H ratio is 1, and that 1, 2 and 3 are the most commonly used proportions in urban design practice [35]. As shown in these examples, the exact optimal D/H ratios claimed by theorists are somewhat different, but all are smaller than the 3/1 ratio that Lynch proposed. The D/H ratio principle is widely used in practice and research, but theorists have not provided sufficient empirical evidence for the optimal D/H ratios for the same reasons that other urban design theories are challenging to prove—it is difficult to test theories using precisely controlled experiments in real urban environments.

Some researchers have empirically tested theorists’ arguments about D/H ratio to find an optimal value. Im applied a traditional “block game approach” using 1:200 scale wood blocks to find the optimal D/H ratio in terms of sense of human scale in urban squares, and found that 4.26 is ideal for laypersons [36]. He also conducted free response survey for urban design experts, and revealed that they consider 2.55 the most ideal value for the same question [36]. However, both approaches have failed to provide a realistic example of each D/H ratio for the respondents. To address this limitation, his earlier research has used visual aids. Im applied a photographic evaluation approach, and suggested that university students and visitors perceived that 6.67 is the optimal D/H ratio for the courtyards enclosed by dormitory buildings [37]. Im also applied simulation-based perspective drawings with a ratio between 1/2 to 20, and found that for the courtyard spaces enclosed by three-, five-, and twelve-story buildings, the maximum visual preference occurred at the D/H ratio of 9.60, 6.95, and 5.12, respectively [9]. Using advanced computer simulation techniques, subsequent studies adopted more realistic urban spaces, even including detailed building façade design, and revealed that 1.33 is ideal [38]. More recently, Kim conducted an experiment to test the D/H ratio principle with computer-generated images of enclosed urban public squares [10]. However, the experiment was limited because the simulated images could only cover a limited viewing angle (specifically, vertical). As a result, squares with a D/H ratio smaller than 2/1 could not be simulated; therefore, only squares with D/H ratios larger than 2/1 were tested in the experiment. Because of such a technical limitation of the simulated images, the study assumed a linear relationship between the D/H ratios and the perceived qualities of an urban public square and tested only the statistical significance of the linear relationship, not whether the theoretically optimal ratio coincided with an optimal reality (p. 14 [10]). In addition, although using advanced 3D modeling techniques, the study projected simulated models onto 2D planes to conduct a visual assessment survey, thereby leading to a lack of realism.

However, in the VR environment, a user can observe the surrounding spaces without the limitations of viewing angles. Using these advantages, this study conducted an experiment to test the validity of the D/H ratio principle empirically by comparing the optimal D/H ratios suggested by urban design theorists with optimal values chosen by users in VR. In the following section, the authors explain the design and process of the experiment.

## 3. Experiment Design and Analytical Framework

### 3.1. Research Questions for the Experiment

The main purpose of this experiment is to test whether the optimal D/H ratios proposed by theorists for the design of urban public squares are consistent with the optimal D/H ratios observed in this experiment (i.e., the D/H ratios that people perceive as being optimal in a virtual space). Therefore, the main research question for the experiment is: “Are the D/H ratios that people perceive to be appropriate for an urban public square and the theoretical optimal D/H ratios proposed by urban design theorists the same?”

### 3.2. Design of the Experiment

To address the research question, the study conducted a visual assessment survey with computer-generated and immersive VR square models. That is, the authors let the participants experience various enclosed urban public squares simulated by computer graphics with a head-mounted device (HMD) and asked them to assess the perceived qualities of the squares. For this experiment, the authors developed a VR simulation of eight urban public squares enclosed by buildings. The following section talks about the details and processes of: (1) the 3D modeling and conversion of urban public squares into VR, (2) the visual assessment surveys, and (3) the variables (independent, control, and dependent) of the statistical analysis.

#### 3.2.1. 3D Modeling and VR Conversion

The authors used SketchUp Make 2016 (Trimble Inc., Sunnyvale, CA, USA) for the 3D modeling of the squares, and eight urban public squares with different D/H ratios were modeled. The sizes of all eight squares were 50 m × 50 m, which are human-scale squares that can support diverse types of social interactions. Gehl proposed that “we usually recognize the person at somewhere between 50 and 70 m…at a distance of about 22–25 m, we can accurately read facial expression and dominant emotions” ([7], p. 34). Therefore, in a square of this size (50 m × 50 m), a person can experience diverse degrees of social interactions, such as just recognizing someone, observing others’ activities, and communicating closely with each other. D/H ratios of the squares were decided by the heights of the surrounding buildings.

**Figure 1:**
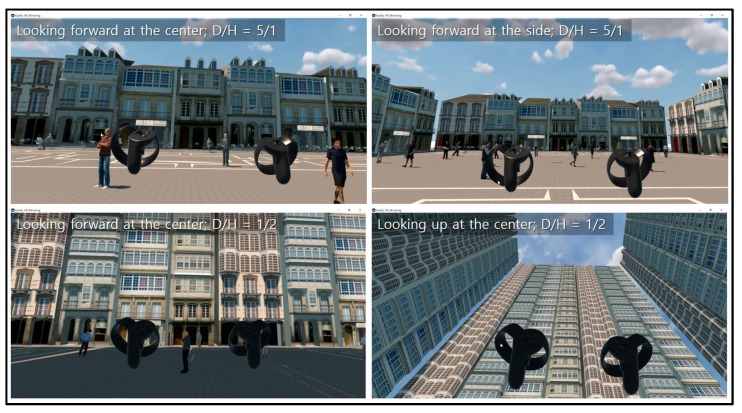
Sample images of simulated squares (captured images of Kubity VR mirroring).

The façades of the surrounding buildings were modeled in a European classic style for two reasons: First, the influential urban theorists mentioned in this paper, such as Lynch and Sitte, lived in Western cities and were familiar with the Western classic-style building facades. The authors assumed that the urban theorists might have these façades in mind when they proposed their optimal D/H ratios. The authors therefore modeled the European classic-style building facade for the purpose of fair comparison. Second, enclosed urban squares themselves originated from European countries. Squares surrounded by European-style buildings are familiar even to Koreans who have not visited European cities, because they have been exposed to Western culture and have experienced such squares via media such as TV and movies. In addition, 36 people (26 standing and 10 sitting) were added to each square to provide a reference for scale and increase the reality of the scene. For VR conversion of the simulated square models, the authors used Kubity (https://www.kubity.com/), a program that converts 3D models from SketchUp (Trimble Inc., Sunnyvale, CA, USA) and Revit (Autodesk, Inc., San Rafael, CA, USA) into VR and allows users to experience the VR converted 3D models at eye level (Figure 1). 

**Figure 2:**
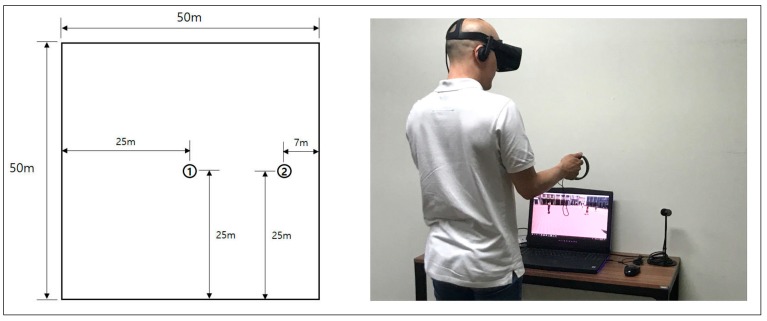
Observation points (center ① and side ②) (**Left**) and actual VR experiment scene (**Right**).

The authors used the Oculus Rift (Oculus, Menlo Park, CA, USA) with Oculus Touch controllers (Figure 2, right) as the HMDs.

#### 3.2.2. Process of the Visual Assessment Survey with VR

The authors recruited experiment participants from the students majoring in urban planning at two universities in the Seoul metropolitan area (G and C universities). A total of 69 participants were recruited through voluntary applications. In the experiment using the VR device (HMD), only one person could participate at a time, and hence experimenting with multiple participants was difficult. In addition, because of the limitation in the number of participants, a homogeneous group of participants (i.e., Korean university students majoring in urban planning) was more relevant to the experiment than a heterogeneous one would be; with a limited number of participants, experiment results from heterogeneous participants would be difficult to properly interpret. For the same reason, Schwebel et al. also conducted VR-related experiments with a small number of homogeneous participants (i.e., 47 elementary school children) ([39], p. 10).

The experiment was conducted individually for each participant with an instructor for guidance, because most participants were not familiar with the VR device (HMD). The instructor first explained to each participant how to wear the HMD and use the controllers to navigate in the VR space before the participant put on the HMD. Then, the instructor asked the participants to carry out the experiment in the following order while wearing the HMD: (1) enter one of the eight squares in the order determined by a random number table; (2) move to the center of the square (point ① of Figure 2) and look around for 30 s; (3) move to its side area (point ② of Figure 2) and look around for 30 s again (points ① and ② are marked on the surface of each square in the shape of a manhole for the participants to locate easily); (4) answer the instructor’s questions about their assessment of the square using a seven-point Likert scale (The participants were first informed that “1” means “strongly disagree,” “2” means “disagree,” “3” means “somewhat disagree,” “4” means “neutral,” ”5” means “somewhat agree,” ”6” means “agree,” and “7” means “strongly agree.” They were then asked to express their opinions using the numbers between 1 and 7 for the statements describing the eight qualities of the simulated squares, such as “The square has a sense of openness,” “The square is cozy and comfortable,” or “The square is suitable for informal social activities, such as meeting with friends and a chat with neighbors.”); (5) move to another square in the next order and repeat the same process for all eight squares.

### 3.3. Variables

#### 3.3.1. Independent Variables

This experiment focuses on the influence of D/H ratios on the perceived qualities of urban public squares. Therefore, D/H ratios were set as independent variables. The authors determined the range of D/H ratios for the experiment using Lynch’s proposed optimal D/H ratios for an urban public square [6]. Therefore, the authors included the proposed D/H ratios of 3/1 and 2/1 and added three more D/H ratios larger than 3/1 (6/1, 5/1, and 4/1), as well as three more D/H ratios smaller than 2/1 (1/1, 1/2, and 1/3).

#### 3.3.2. Control Variables

Social and urban studies generally include basic socio-demographic variables, such as gender, age, race, income, job, and education, as control variables [40,41,42,43]. However, among these, variables other than gender and education (i.e., age, race, income, and job) did not need to be included in the control variables, because all participants were Korean university students in their 20s, and therefore such control variables were not relevant. Instead, the authors included other variables expected to affect participants’ assessment of the squares, such as university, grade level, overseas travel experience, and large city residence. The variables “university” and “grade” are related to education, and the relationship between education and visual preference has been tested in many urban design and landscape architecture studies [44,45,46]. The university variable was included because participants were recruited from two universities and the differences in their curricula might influence the results of the experiment. The grade level variable was included because the level of education for urban planning and urban design might influence participants’ responses. In particular, first and second-year students were classified as lower grade, and the rest were classified as upper grade. The variables “overseas travel experience” and “large city residence” are related to the familiarity of the environment being studied; the relationship between familiarity (and experience) and visual preference has also been examined in many studies [43,44,47]. For the variable “overseas travel experience,” the authors classified the participants’ travel experiences according to the period of travel in Western cities, because urban public squares enclosed by buildings are a more common type of public space in Western cities than in Oriental cities. For the variable “large city residence,” participants were classified according to the population of the city where they had lived for the longest period, because enclosed urban public squares are more common in large cities than in smaller ones [10]. With the exception of the university variable, most of the control variables were the ones applied in Kim’s [10] previous study (Table 1).

#### 3.3.3. Dependent Variables: Perceived Qualities of Enclosed Urban Squares

Instead of using the participants’ assessment of the degree of enclosure as a dependent variable, this study used their assessment of the intuitive qualities of an enclosed urban public square and its behavioral suitability for optional and social activities similar to Kim’s previous study [10]. “Sense of enclosure” has been suggested or implied as the major goal when urban design theorists have proposed optimal D/H ratios [6,26]. However, a sense of enclosure is an obscure concept that is difficult for non-experts to understand. Moreover, the enclosure of an urban public square is a tool for designing the square as a good place, rather than the ultimate goal itself ([10], p. 6).

This study employed the eight variables used in Kim’s study [10] as dependent variables. These were participants’ assessment of the five intuitive qualities of public squares—openness, magnificence, coziness, dauntingness, and overall goodness—and an evaluation of the behavioral suitability of the square for three types of optional and social activities that are likely to happen in a good urban public square (Table 2). Regarding the relationship between urban spaces and people’s activities, Gehl classified activities into necessary/functional, optional/recreational, and social activities, and claimed that urban spaces of high quality promote optional and social activities ([7], p. 21). Reflecting Gehl’s claim for good urban spaces, the authors included optional activities and social activities as dependent variables, not including necessary activities. Regarding social activities, the environments suitable for informal social activities and those for formal social activities might be different, and they need to be distinguished; therefore, the authors divided them into informal and formal social activities.

### 3.4. Statistical Model for Data Analysis: Quadratic Regression Model

As a statistical model to analyze the results of the experiment, the authors chose the quadratic regression model with maximal or minimal values. The model choice was mainly based on the existing D/H ratio principle assuming that there is an optimal D/H ratio for the design of an enclosed urban public square. In the following section, the authors explain the descriptive statistics, conduct the quadratic regression analyses, and compare the results of the analyses with the optimal D/H ratios from the existing theories.

## 4. Analysis of the Results

### 4.1. Descriptive Statistics

The research design originally consisted of eight statistical analyses of 552 records for each quality (69 participants × 8 squares in VR); therefore, 4416 values (8 analyses × 552 records) should have been collected. However, during the experiment, seven records were missing, and eventually 4409 valid values were used for the analysis. Table 3 illustrates the percentages and numbers of participants for each control variable. As illustrated, the participants were evenly selected from both universities and gender groups.

**Figure 3:**
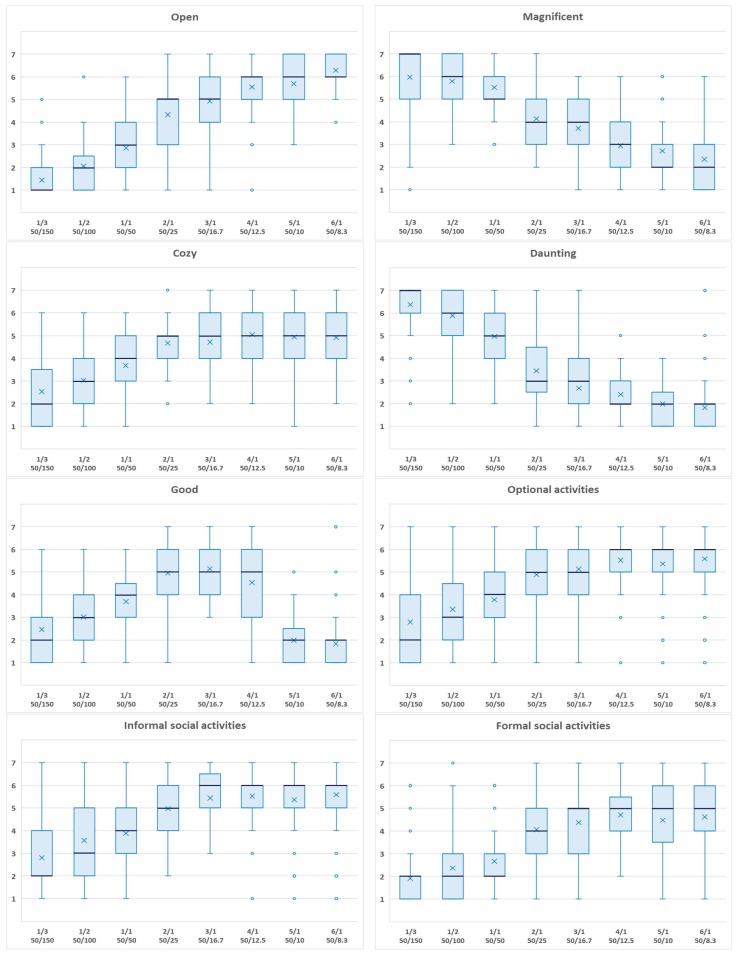
Box-and-whisker plots of the scores for eight dependent variables by D/H ratio.

Table 4 illustrates the means and standard deviations of participant scores (seven-point Likert scale) for eight dependent variables by D/H ratio, and Figure 3 illustrates the box-and-whisker plots of the scores for eight dependent variables by D/H ratio. In Table 4 and Figure 3, the results for six dependent variables, not including magnificence and dauntingness, roughly illustrate that participants provided higher scores for squares with higher D/H ratios, which implies that the optimal D/H ratios for these six qualities of the square might be larger than the theoretical optimum of 3/1 and 2/1.

The interpretation of Table 4 and Figure 3 described above is preliminary, without any control for the respondents’ personal characteristics. Therefore, for a more precise interpretation, the authors conducted quadratic regression analyses based on the existing D/H ratio principle assuming that an optimal D/H ratio for the design of an enclosed urban public square exists.

### 4.2. Quadratic Regression Analyses

**Figure 4:**
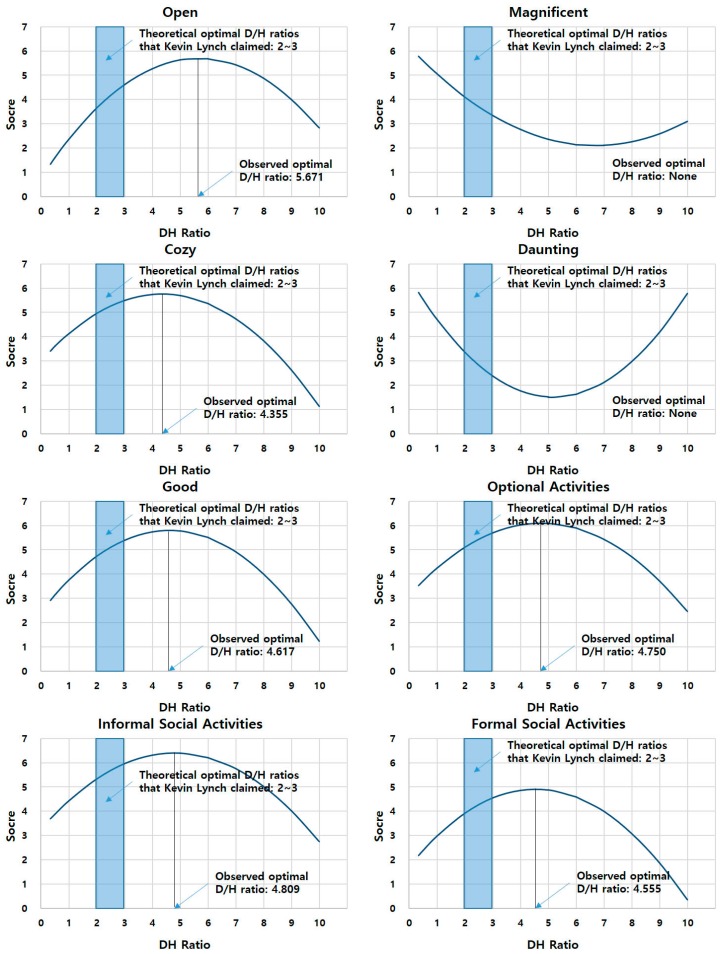
The relationship between D/H ratios and the eight dependent variables.

The objective of the quadratic regression analyses was to address the research question of this study: that is, “Are the D/H ratios that people perceive to be appropriate for an urban public square and the theoretical optimal D/H ratios proposed by urban design theorists the same?” For this purpose, the authors examined the statistical significances of the models, calculated the optimal D/H ratios that maximize the value of the dependent variables in the experiment, and compared these with the optimal D/H ratios put forward by theorists. Table 5 illustrates the results of the analyses for the eight dependent variables. The five models in Table 5 are for the five intuitive qualities of the squares (i.e., openness, magnificence, coziness, dauntingness, and overall goodness) and the three models are for the behavioral suitability of the square for optional and social activities (i.e., wandering individually, gathering informally with friends, and holding formal events). The models estimated the coefficients of the test variables by controlling the characteristics of the participants. The graphs in Figure 4 visualize the relationships between D/H ratios and the eight dependent variables on the basis of the results of the quadratic regression analyses (i.e., the coefficients in Table 5) (to draw the graphs, the authors input “0” for the dummy control variables and the mean value for the continuous control variable). In particular, the authors compared the observed optimal D/H ratios with the optimal D/H ratios that Lynch claimed: that is, those between 2/1 and 3/1.

First, regarding the statistical significance of the models, all eight quadratic regression models in Table 5 illustrate that the D/H ratio is significantly associated with the eight dependent variables at the 0.01 level. Second, regarding the optimal D/H ratio, in the six models for openness, coziness, goodness, and suitability for all three activities, the D/H ratio and the D/H ratio squared were positively and negatively associated with the dependent variables, respectively. This means that the relationships between D/H ratios and the six perceived qualities of the squares seem to be concave curves with the maximum value. The authors calculated the D/H ratios that maximize the values of the six dependent variables using the coefficients of both test variables. The optimal D/H ratios maximizing the respondents’ scores for openness, coziness, goodness, and suitability for optional, informal social, and formal social activities were 5.671, 4.355, 4.617, 4.750, 4.809, and 4.555, respectively. All six optimal ratios from the experiment were larger than 3/1 or 2/1, the theoretical optimal D/H ratios that Lynch and other theorists proposed. Results suggest that when the height of surrounding buildings is lower than the optimum suggested by theorists, people might feel that an urban public square is more open, cozier, better, and more suitable for optional and social activities.

For magnificence and dauntingness, the relationships between D/H ratio and the dependent variables were convex curves with the minimum value, and the D/H ratios that minimize the participants’ scores for magnificence and dauntingness were calculated as 6.699 and 5.179, respectively. Results contradict the hypothetical premise that an optimal D/H ratio exists that creates the best perceived qualities of an enclosed urban public square. People may perceive the magnificence and dauntingness of squares in a different way than they perceive the other six qualities of the squares.

Some control variables also demonstrated significant relationships with the intuitive qualities and behavioral suitability of the squares. In particular, with the variable “grade,” the six models demonstrated consistent patterns. Upper grade students (juniors and seniors, as well as graduate students) tended to report lower scores than lower grade students for magnificence, coziness, overall goodness, and the behavioral suitability for all three types of activities (Table 5). This might be because when students take more urban design courses, they have a higher standard for evaluating the quality of a public square.

For other control variables, no consistent pattern at a significant level was observed among the eight models. For the university variable, participants from G University tended to report higher scores for openness than those from C University. For the travel experience variable, participants who had traveled in Western cities for longer than 6 months tended to report higher scores for magnificence than those who had not traveled overseas. Participants who had traveled in Western cities for less than 6 months tended to report lower scores for the suitability of the squares for optional activities than those who had not traveled overseas. Participants who had traveled only in Oriental cities tended to report higher scores for magnificence and dauntingness than those who had not traveled overseas.

For the variable “evaluation order,” the order of evaluating the eight squares was randomized for each participant, and therefore an insignificant relationship was expected. However, in the model for “the suitability of the square for informal social activities,” the variable was significantly and negatively associated with the dependent variable. The model demonstrates the tendency of respondents to provide lower scores later in the evaluation. Nonetheless, as only one out of the eight models demonstrated this discrepancy, it would be reasonable to regard it as a coincidence rather than an experimental design error.

## 5. Discussion

This discussion section particularly focuses on the following two issues: (1) the possible causes of the discrepancy between the theoretical optimal D/H ratios and the observed ratios in the experiment; and (2) the appropriateness of suggesting one global model integrating the subdivided results for the eight dependent variables.

**Figure 5:**
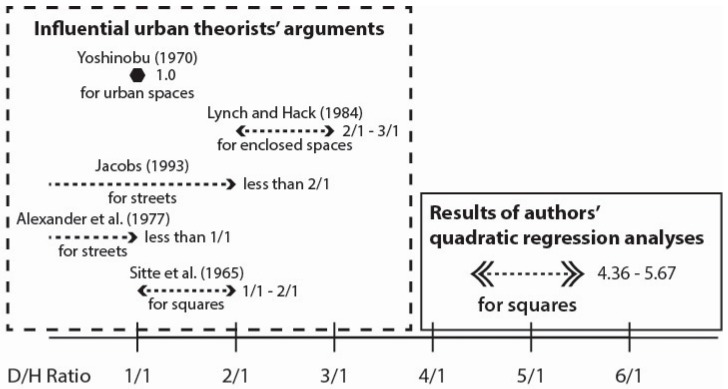
Comparison of optimal D/H ratios: theorists’ arguments and results of authors’ quadratic regression analyses.

Regarding the first issue, Figure 5 summarizes theorists’ arguments and results of our quadratic regression analyses regarding the optimal D/H ratios of enclosed urban spaces. As shown in Figure 4 and Figure 5, the optimal D/H ratios for the six perceived qualities of a square (openness, coziness, goodness, suitability for optional activities, suitability for informal social activities, and suitability for formal social activities) are around four to six. On the contrary, the theoretical optimal D/H ratios proposed by influential urban theorists including Lynch [6] are mostly below three, much lower than the results of our experiments. We can speculate the following possible causes for this discrepancy. First, it might be due to different aesthetical preferences between experts and laypersons. Scholars have argued that experienced experts and laypersons experience and evaluate the qualities of buildings and spaces differently [48,49]. For example, Mohammad et al. demonstrated the different aesthetical preferences between architects and non-architects in residential façade design [48] and Robert et al. investigated the differences between them in experiencing large contemporary buildings. Im also found that urban design experts prefer lower D/H ratio (i.e., higher buildings) to laypersons [36]. If the same cause is applicable to this study, laypersons’ perception should be considered more carefully because the main users of public spaces including urban squares are not experts but laypersons. Of course, further experiments involving both experts and laypersons should be conducted in order to determine whether the differences in perception between experts and laypersons actually affected the results of this study. Second, the difference of the times might be another possible cause of the discrepancy. That is, while the urban theorists who proposed the optimal D/H ratios lived in the period between the end of the nineteenth century and the middle of the twentieth century, the participants of this experiment are living in the 21st century. Because of the improvement in mobility and the development of media and communication technology, the latter might have experienced more diverse urban spaces directly or indirectly than the former, which might have changed people’s perception. If this is the case, the optimal D/H ratio might need to be updated. Third, this discrepancy might be due to the limitations of the exploratory nature of this study. For instance, as an exploratory study, this study tested the D/H ratios in a specifically simulated environment, that is, squares with a fixed size (50 m × 50 m) and one type of façade design (European classic style). As the authors explained in Section 3.2.1, they modeled the squares in this way for the sake of fair comparison between the theoretical D/H ratios and the observed ones. However, we cannot rule out the possibility of coincidence between the theoretical ratios and the observed ones in other contexts, that is, squares having other conditions. Future studies on diverse types of squares that have different conditions, such as different sizes and façade designs, are needed to test such a possibility.

The second issue to discuss is the appropriateness of suggesting one global model integrating the subdivided results for the eight dependent variables. Regarding this issue, the authors originally assumed that the optimal D/H ratios for the square might vary according to the social functions needed in each context. For example, the optimal D/H ratio for a cozy community square for informal social meetings might be different from that for a magnificent square for formal national or religious ceremonies. That is, it might be more appropriate to find multiple optimal D/H ratios for different contexts. Therefore, the authors asked the participants questions about the subdivided qualities of space. However, results of the experiments showed similar patterns for some of the qualities, if not for all. Therefore, it might be possible to propose sub-global models that integrate several qualities, if not one global model for all qualities.

## 6. Conclusions

Using an immersive VR technique, this study empirically examined a well-known principle about urban public spaces: the D/H ratio principle. The authors conducted an experiment using VR to address the question of whether the optimal D/H ratios that people feel are best for a public square match the optimal D/H ratios claimed by theorists for public square design. Specifically, the authors divided the qualities of an enclosed urban public square into the following eight categories: openness, magnificence, coziness, dauntingness, overall goodness, and suitability for three types of optional and social activities. For the statistical analyses of the experiment results, the authors used quadratic regression models.

The analyses suggest that, except for magnificence and dauntingness, the optimal D/H ratios observed from the experiment for the six qualities are larger than those based on theory. This suggests that people might feel that a square surrounded by buildings shorter than those claimed by theorists as optimal is more open, cozier, better, and more suitable for optional and social activities. Regarding magnificence and dauntingness, the results are different from the hypothetical premise that there exists an optimal D/H ratio maximizing the qualities of a public square. People might feel the magnificence and dauntingness of a public square in different ways than they do qualities such as coziness and suitability for social activities; they might feel that a square is less magnificent and less daunting as the D/H ratio increases.

This study contributes to the urban design field by first demonstrating a way in which immersive VR can be utilized to test existing urban design theory and overcome the limitations of existing studies based on 2D visualization methods. This includes overcoming the problems of viewing angles and the visual distortion created by projecting 3D models onto 2D planes.

The study also has the following limitations that need to be addressed in future research: (1) Other design characteristics, such as the size of the public square and design of building façades may affect the experimental results, including the optimal D/H ratio, as Kim suggested in previous research [10]. For example, the optimal D/H ratios for larger squares (e.g., 200 m × 200 m) might differ from the optimal ratios observed in this study. It would be the same for the façade design of surrounding buildings. It would be more reasonable to have different optimal D/H ratios for different contexts. Therefore, future studies might need to be conducted in diverse contexts to find optimal D/H ratios for each context. (2) The experiment was conducted with 69 students from two universities majoring in urban design and planning. This was a method chosen deliberately to find homogeneous groups because the nature of this study, which requires the use of HMDs, makes it difficult to experiment with many participants. However, the degree of familiarity with the urban design field might influence the experiment results, as the authors mentioned in the discussion section. Even in this study, the participants’ level of training in the field of urban design (grade level variable) had a significant effect on most of the dependent variables. That is, the responses of general citizens might differ from those of experiment participants. Thus, to generalize this study’s findings, additional experiments with general citizens are needed in the future. (3) Lastly, the experiment of this study was conducted only with Korean participants. Although the participants would be familiar with urban squares surrounded by European-style buildings because they have experienced Western cities and cultures directly or indirectly through oversea travel or media, the participants’ responses might differ from those of Western citizens living in Western cities. Therefore, the results of this study should not be overly generalized and future research on Western citizens is needed to overcome this limitation.

## Figures and Tables

**Table 1:** Control variables, data coding, and reference category.

Variables	Data Coding	Reference Category
University	G University C University	C University
Grade	Upper grade (juniors, seniors, and graduate students) Lower grade (first and second grade)	Lower grade
Overseas travel experience	Traveled in Western cities for longer than six months Traveled in Western cities for less than six months Traveled only in Oriental cities No overseas travel	No overseas travel
Large city residence (population)	Large cities (over 1,000,000) Medium cities (between 500,000 and 1,000,000) Small cities (under 500,000)	Small cities
Gender	Male Female	Female

**Table 2:** Classification of dependent variables.

Classification	Dependent Variables
Intuitive qualities of the square	Open Magnificent Cozy Daunting Good ^a^
Behavioral suitability of the square for optional and social activities	Optional activities: wandering individually Informal social activities: gathering informally with friends Formal social activities: holding formal events, such as festivals or political rallies

^a^ Overall assessment of the four dimensions of open, magnificent, cozy, and daunting.

**Table 3:** Descriptive statistics of control variables.

Control Variables		Percentage of Participants	Number of Participants
University	G University	56.5%	39
	C University (reference group)	43.5%	30
Grade	Upper grade	55.1%	38
	Lower grade (reference group)	44.9%	31
Gender	Female	55.1%	38
	Male (reference group)	44.9%	31
Countries traveled	Traveled in Western cities for longer than six months	13.0%	9
	Traveled in Western cities for less than six months	30.4%	21
	Traveled only in Oriental cities	49.3%	34
	No overseas travel (reference group)	7.2%	5
Large city residence	Large city (over 1,000,000)	66.7%	46
	Medium city (500,000 ~ 1,000,000)	11.6%	8
	Small city (under 500,000; reference group)	21.7%	15

**Table 4:** Means and standard deviations of scores for eight dependent variables by the D/H ratio.

D/H Ratio (Square Width/Bld. Height)	Intuitive Qualities of the Squares					Behavioral Suitability of the Squares for Optional and Social Activities		
	Open	Magnificent	Cozy	Daunting	Good *^a^*	Optional Activities	Informal Social Activities	Formal Social Activities
6/1 (50 m/8.3 m)	6.290 (0.800)	2.348 (1.295)	4.913 (1.248)	1.826 (1.129)	5.522 (1.162)	5.580 (1.244)	5.826 (0.947)	4.623 (1.543)
5/1 (50 m/10 m)	5.696 (1.171)	2.710 (1.436)	4.942 (1.190)	1.986 (1.097)	5.464 (1.098)	5.362 (1.340)	5.667 (1.002)	4.478 (1.547)
4/1 (50 m/12.5 m)	5.551 (1.084)	2.942 (1.238)	5.043 (1.135)	2.406 (0.983)	5.358 (1.018)	5.522 (1.187)	5.667 (0.912)	4.710 (1.275)
3/1 (50 m/16.7 m)	4.928 (1.266)	3.710 (1.395)	4.710 (1.205)	2.681 (1.268)	5.145 (1.195)	5.130 (1.413)	5.485 (1.254)	4.377 (1.415)
2/1 (50 m/25 m)	4.333 (1.421)	4.130 (1.262)	4.667 (1.112)	3.449 (1.593)	4.957 (1.313)	4.884 (1.547)	4.971 (1.329)	4.058 (1.559)
1/1 (50 m/50 m)	2.855 (1.277)	5.522 (1.030)	3.681 (1.324)	4.971 (1.154)	3.696 (1.208)	3.783 (1.493)	3.884 (1.430)	2.667 (1.175)
1/2 (50 m/100 m)	2.058 (1.238)	5.797 (1.174)	3.029 (1.434)	5.884 (1.136)	3.014 (1.245)	3.362 (1.633)	3.565 (1.681)	2.362 (1.424)
1/3 (50 m/150 m)	1.435 (0.859)	5.971 (1.532)	2.536 (1.358)	6.377 (0.994)	2.464 (1.400)	2.797 (1.656)	2.812 (1.525)	1.913 (1.139)

*^a^* Overall assessment of the four dimensions of open, magnificent, cozy, and daunting.

**Table 5:** Quadratic regression estimates on the intuitive qualities of the public squares and their behavioral suitability for optional and social activities.

Variables	Intuitive Qualities of the Square					Behavioral Suitability of the Square for Optional and Social Activities		
	Open	Magnificent	Cozy	Daunting	Good ^c^	Optional Activities	Informal Social Activities	Formal Social Activities
D/H ratio	1.734 **	−1.218 **	1.263 **	−1.906 **	1.455 **	1.253 **	1.307 **	1.403 **
D/H ratio squared	−0.153 **	0.091 **	−0.145 **	0.184 **	−0.158 **	−0.132 **	-0.136 **	−0.154 **
G university	0.253 *	0.109	−0.137	−0.091	0.027	−0.191	−0.089	−0.022
Upper grades	−0.084	−0.311 **	−0.392 *	−0.080	−0.438 **	−0.225	−0.314 **	−0.341 **
Female	−0.042	0.052	−0.084	0.054	−0.050	0.001	−0.182	0.157
Travel experience ^a^								
In Western cities for longer than 6 months	0.343	0.620*	0.042	0.339	0.450	−0.050	0.010	−0.008
In Western cities for less than 6 months	0.137	0.217	−0.350	0.277	−0.003	−0.575 *	−0.470 *	−0.144
Only in Oriental cities	0.361	0.470 *	−0.127	0.650 **	0.352	−0.123	−0.219	0.195
City size ^b^								
Over 1,000,000	0.002	0.131	−0.170	0.062	−0.067	0.014	0.015	−0.159
500,000~1,000,000	0.391	0.057	0.164	−0.117	0.012	0.297	0.209	−0.111
Evaluation order	−0.002	−0.031	−0.002	−0.011	−0.021	−0.011	−0.060 *	0.010
Constant	0.783 **	6.180 **	3.006 **	6.441 **	2.445 **	3.124 **	3.533 **	1.717 **
No. of observation	552	552	552	552	550	552	551	552
Adjusted *R*-square	0.675	0.525	0.345	0.668	0.467	0.328	0.420	0.362
D/H ratio that maximizes/minimizes the value of the dependent variable	5.671	6.699	4.355	5.179	4.617	4.750	4.809	4.555

^a^ The reference category for travel experience is “No overseas travel”; ^b^ The reference category for city size is “Under 500,000”; * p ≤ 0.05 two-tailed; ** p ≤ 0.01 two-tailed; ^c^ Overall assessment of the four dimensions of open, magnificent, cozy, and daunting.
